# An Enlarged Superior Horn of Thyroid Cartilage Presenting During Intubation: A Case Report

**DOI:** 10.1155/cria/6068920

**Published:** 2026-08-23

**Authors:** Colin Bashline, Peter Herbig

**Affiliations:** ^1^ West Virginia School of Osteopathic Medicine, 400 Lee St North, Lewisburg 24901, West Virginia, USA, wvsom.edu; ^2^ Raleigh General Hospital, 1710 Harper Road, Beckley 25801, West Virginia, USA

## Abstract

**Introduction:**

Variations in laryngeal anatomy, particularly of the superior horn of the thyroid cartilage (SHTC), can pose challenges in airway management. While minor anatomic differences are commonly encountered, clinical encounters of SHTC variants are rarely documented. We present a case of an asymptomatic SHTC variant identified during routine intubation, the first documented with high‐definition video.

**Case Presentation:**

A 55‐year‐old male presented for a sacroiliac fusion operation. He reported a prior intubation during which clinicians noted an abnormal laryngeal structure. Preoperatively, he denied dysphagia, phonation changes, or respiratory symptoms, though he did describe intermittent throat “tightness.” Intubation was performed with video laryngoscopy, which revealed a prominent SHTC without impediment to endotracheal tube placement. The procedure and extubation were uneventful. Postoperative review of prior imaging demonstrated the variant anatomy on a cervical CT scan obtained 23 years earlier, predating anterior cervical fusion surgery, and on a lateral cervical radiograph from 4 years prior.

**Discussion:**

SHTC variants are uncommon and often asymptomatic, though patients may report foreign body sensation, odynophagia, or neck discomfort. They are often associated with prior neck trauma, and age‐related ossification increases fracture risk. Literature documents occasional airway management complications, including failed laryngeal mask airway placement. Consideration of anatomic variants should be in the differential diagnosis, as they may support avoidance of supraglottic airway devices in these patients. Management ranges from observation to specialist referral for symptomatic or severe cases.

**Conclusion:**

Anomalous SHTC morphology is a rare finding with implications for airway management. Clinicians should include thyroid cartilage variants in the differential diagnosis of patients with unexplained throat symptoms and remain vigilant during intubation. Further research is warranted to better define the prevalence, clinical impact, and management strategies for atypical thyroid cartilage anatomy.

## 1. Introduction

Intubating clinicians are trained to anticipate and navigate a certain degree of variability in human anatomy. Numerous ossified, cartilaginous, and soft tissue structures comprise the pharynx and larynx. A small collection of studies have evaluated the variation present in the major structures; the largest being the thyroid cartilage [1–3]. The thyroid cartilage is comprised of two laminae, joining at an angle on their anterior ends [4]. On the posterior end, a superior and inferior horn protrude from the main body, connecting to the hyoid bone and cricoid cartilage, respectively [5]. The superior horn of the thyroid cartilage (SHTC) is known to be variable, but documentation has been limited to case reports [6–8]. Identification of these variant structures relies on careful assessment of cervical imaging or laryngoscopic visualization, making preoperative identification challenging. The deep nature of these structures also prevents their identification with noninvasive preoperative evaluation, such as the Mallampati score. While documentation of the clinical impacts of these variants is limited, the changes in anatomy pose a possible modality of failure for airway management, specifically with use of supraglottic airways [9]. We discuss a case of a patient with a SHTC variant significant enough to be discovered in our routine clinical practice and believe that we are the first to record one of these variants on video.

## 2. Case Presentation

We present the case of a 55‐year‐old male patient who presented to the anesthesia service for anesthetic management for a sacroiliac fusion operation. Through the course of the preoperative interview, the patient mentioned that following a previous operation, an abnormal structure had been noted by intubating clinicians. The structure did not create any complications during the intubation, but the patient was instructed to mention the variation to future providers. The patient did not recall a history of neck trauma or major trauma in general beyond a previous anterior approach cervical vertebral fusion operation. The patient was minimally symptomatic, with no changes in phonation, difficulty breathing, or history of aspiration noted. No pain or difficulty swallowing were noted prior to presentation, but when asked, the patient had noted an intermittent sensation of “tightness” in his throat that had not been discussed with a physician previously. Whether this sensation of tightness was related to the anatomic variation is unknown, and the patient did not recall having this sensation to a bothersome degree in the past.

Preoperative screening determined the patient’s American Society of Anesthesiologist’s (ASA) Class to be 3 due to a history of GERD, hypercholesterolemia, hypertension, and OSA. His Mallampati score was 2. No dentures, loose dentition, or abnormal oral anatomy were noted. Due to previous successful intubation without notable difficulty and lack of available in‐house ear, nose, and throat (ENT) physicians, preoperative ENT consultation and further preoperative review of imaging were deferred, and senior anesthesiology staff arranged to be present to monitor any possible complications with airway management.

**FIGURE 1:**
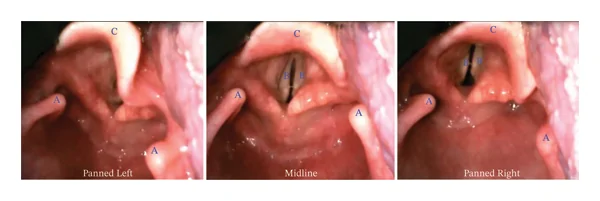
Snapshots showing the laryngoscope view in midline and panned left and right. A = superior horn of thyroid cartilage, B = vocal cords, and C = epiglottis.

**FIGURE 2:**
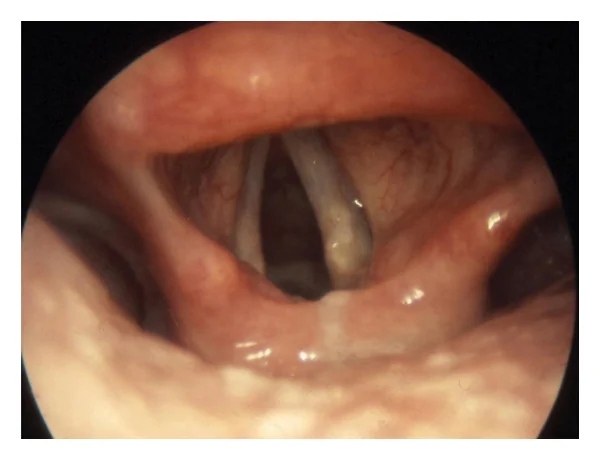
Image showing classic laryngeal anatomy when viewed by laryngoscope [10].

With the advanced notice of variant anatomy, the patient consented to a recording being taken of his intubation using the built‐in video recorder function of our video laryngoscope. The patient was sedated and paralyzed with 50 mcg fentanyl, 100 mg lidocaine, 2 mg midazolam, 200 mg propofol, and 120 mg succinylcholine via peripheral intravenous line. The patient was maintained under general anesthesia with sevoflurane, propofol, and fentanyl. Intubation was performed by a certified registered nurse anesthetist (CRNA) using a video laryngoscope and an 8 mm endotracheal tube. Prior to tube placement in the glottic opening, the video scope was panned left and right to evaluate the anatomy (Figure 1). For comparison, an example of classic laryngeal anatomy has been provided as well (Figure 2) [10]. The anatomic structures did not impede the path of endotracheal tube placement. Endotracheal intubation proceeded without added difficulty. The patient underwent their scheduled operation and was extubated without issue. Postoperatively, no adverse outcomes were noted beyond a sore throat and operative site pain. Due to largely absent symptomatology, the patient did not express interest in ENT physician assessment or pursuit of treatment.

**FIGURE 3:**
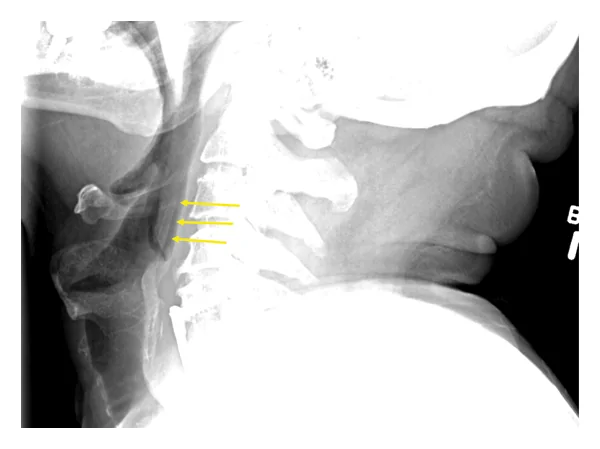
Lateral cervical X‐ray demonstrating the elongated superior horn of the thyroid (yellow arrows).

**FIGURE 4:**
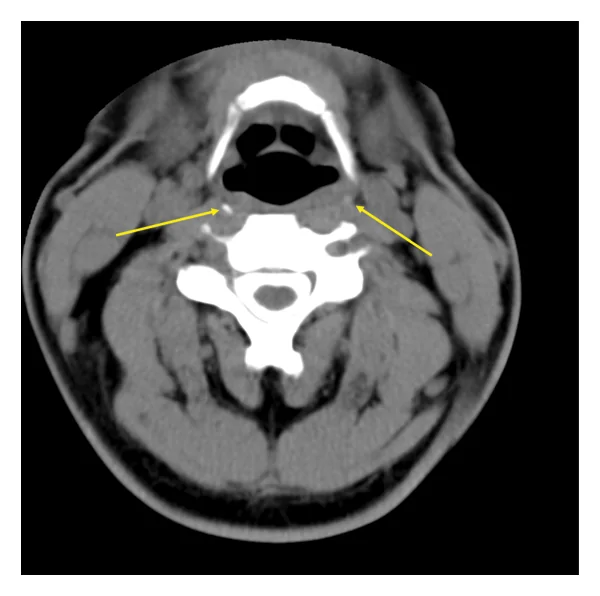
Axial CT scan demonstrating the superior horn of the thyroid cartilage (yellow arrows) at the approximate level of the vallecula.

Follow‐up consisted of evaluating previous imaging. A lateral cervical X‐ray from 4 years prior revealed a prominent SHTC (Figure 3). This prominence was further characterized by evaluation of a CT scan from 23 years prior (Figure 4). Importantly, the CT was performed prior to the cervical fusion, suggesting that the anatomy was present prior to operation.

## 3. Discussion

Cases of variations in thyroid cartilage have been noted across the literature, with varying degrees of impact on clinical practice. Most patients are relatively asymptomatic, with the most common presenting symptom being a foreign body sensation in the throat [6, 11, 12]. However, patients present with odynophagia, neck pain, and coughing fits [13–15]. While the prevalence of these variations is not well characterized, one ENT group performed laryngoscopy on all patients complaining of globus sensation and found that 3% of patients studied had SHTC variants [7]. While some studies suggest a notable prevalence of SHTC variants, these rates are reported from symptomatic patients presenting for specialist care, and publication bias may play a role in the rates seen.

This variant anatomy is often associated with a history of neck trauma of varying severity [14, 16]. Physiologic ossification has sexual variability, occurring in the 40’s and 50’s in men, and in the 70’s in women [4]. After ossification, the superior horn is less flexible and therefore more prone to fracture, making consideration of trauma history even more important in older patients. Fractures of the SHTC have been documented to cause rare but severe outcomes such as mediastinitis or laceration of the carotid artery [14, 16]. Other case reports have documented presentations of abnormal SHTC with no trauma history [6, 17]. However, we believe our case to be one of the few examples of these variants documented with high‐definition photography, and the first documented with video laryngoscopy, allowing for demonstration of how these anatomic structures move when manipulated.

While these are interesting anatomic variants, consideration should be given to the possible clinical implications, particularly in reference to airway management. Documented clinical impact is limited to a single case report. Browning et al. documented an abnormal SHTC variant that prevented the successful placement of a laryngeal mask airway across multiple attempts [9]. Browning and Whittet went on to document multiple variants that presented to their ENT clinic, going as far as to provide a grading system for SHTC variants [7]. In the initial case that Browning et al. described, it was hypothesized that the medial deflection of the SHTC formed a bar that prevented the passage of the tip of the LMA [9]. In our case, the SHTC deflects more superiorly than medially. We hypothesize that this increased occupation of the supraglottic space could interfere with the seal that LMAs form with the glottic opening. While Browning et al. discussed interference with a classic LMA, we also believe that modern supraglottic airways such as i‐gels, AuraGain, and contemporary LMA models (among others) would face similar concerns. Retroglottic devices such as the King Laryngeal Tube and Combitube may not be as impacted as their sealing balloons are in the esophagus and in the pharynx.

Furthermore, the thyroid cartilage acts as the anterior attachment point of the vocal cords; applying pressure to these horns could displace the vocal cords, possibly causing minor difficulty for endotracheal intubation. However, as discussed in Browning et al.’s study, nonsupraglottic methods such as endotracheal intubation or transtracheal ventilation should be considered as an alternate approach when these variants impose difficulty with LMA insertion. Another case report documented endotracheal intubation complicated by abnormal thyroid cartilage, but this difficulty was notably caused by a unilateral osteoma of the SHTC rather than a simple congenital variant [18]. Outside of these two case descriptions, no further discussion of the clinical impacts of thyroid cartilage variation exists to our knowledge. Anesthesiology clinicians should give consideration to thyroid cartilage variants as a possible etiology of LMA failure, as the subtle or asymptomatic nature of these variants coupled with the difficulty of assessing these deep structures without laryngoscopy could obscure their presence until patients are already undergoing airway management. While some cases have documented external thyroid cartilage displacement maneuvers as effective for achieving airway control, these have been associated with fractures of the SHTC [19]. A more permanent treatment is surgical resection, but this tends to be reserved for symptomatic or severe cases, and many patients decline surgical intervention [15]. Further research into the role that thyroid cartilage plays in airway management is warranted to further quantify if variance is predictive of management success or difficulty.

## 4. Conclusion

We present what we believe to be the first example of an elongated SHTC variant documented with video. Anatomic variations in the larynx are an uncommon encounter in clinical practice, yet consideration should be given to the complications they may pose in airway management. Elongated superior horns of the thyroid cartilage can interfere with the supraglottic space, potentially preventing appropriate LMA–glottis sealing. Due to these effects, anesthesia clinicians should have an elevated inclination to utilize endotracheal intubation in patients with known SHTC variants, particularly those who have failed LMA placement in the past. Due to a paucity of research into the clinical impacts of SHTC variation, it is unknown if the presence or absence of variants is predictive of airway success. Outside of airway complications, abnormal superior thyroid horn morphology should be considered in the differential of patients presenting with foreign body sensation, odynophagia, dysphagia, and symptoms of laryngeal irritation. Consideration should be given for ENT referral if patients complain of these symptoms. Patients with severe symptoms or previous airway management difficulty due to thyroid cartilage variants may also benefit from preoperative ENT consultation to assess degree of anatomy variance and implications for airway management methods. Mitigation and treatment approaches vary from support and monitoring to surgical resection depending on patient preference and symptom severity. Certainly, research to better characterize the prevalence, variants, and clinical implications of atypical thyroid cartilage morphologies is needed to better understand this unusual condition.

## Funding

This work was supported by the Senior Friends of Raleigh General Hospital.

## Disclosure

This funder provided support for the project but was not involved in decision to publish, writing, editing, or approval.

## Conflicts of Interest

The authors declare no conflicts of interest.

## Supporting Information

Additional supporting information can be found online in the Supporting Information section.

## Supporting information


**Supporting Information 1** De‐identified recording of the intubation during which we identified the abnormal superior horn of the thyroid. The video was taken using the built‐in recording function on our video laryngoscope. CARE statement: This manuscript has been evaluated to meet CARE case report guidelines. A timeline of patient care events is provided in Table 1.

**Supporting Information 2** CARE checklist.

## Data Availability

Data sharing is not applicable to this article as no datasets were generated or analyzed during the current study.

## References

[1] Beckford N. S. , Rood S. R. , Schaid D. , and Schanbacher B. , Androgen Stimulation and Laryngeal Development, Annals of Otology, Rhinology and Laryngology. (1985) 94, no. 6 Pt 1, 634–640, 10.1177/000348948509400622. 4073745

[2] Kovac T. , Popovic B. , Marjanovic K. et al., Morphometric Characteristics of Thyroid Cartilage in People of Eastern Croatia, Collegium Antropologicum. (2010) 34, no. 3, 1069–1073. 20977105

[3] Pinheiro J. , Cascallana J. L. , Lopez de Abajo B. , Otero J. L. , and Rodriguez-Calvo M. S. , Laryngeal Anatomical Variants and Their Impact on the Diagnosis of Mechanical Asphyxias by Neck Pressure, Forensic Science International. (2018) 290, 1–10, 10.1016/j.forsciint.2018.06.019. 29979976

[4] Meyer-Breiting E. and Burkhardt A. , Tumours of the Larynx: Histopathology and Clinical Inferences, 1988, Springer-Verlag.

[5] Friedrich Paulsen J. W. , Sobotta Atlas of Human Anatomy, 2013, 15th edition, Elsevier.

[6] Karaman E. , Saritzali G. , Albayram S. , and Kara B. , An Unusual Cause of Foreign-Body Sensation in the Throat: A Displaced Superior Cornu of the Thyroid Cartilage, Ear, Nose, and Throat Journal. (2011) 90, no. 6, E22–E24, 10.1177/014556131109000612. 21674457

[7] Browning S. T. and Whittet H. B. , A New and Clinically Symptomatic Variant of Thyroid Cartilage Anatomy, Clinical Anatomy. (2000) 13, no. 4, 294–297, 10.1002/1098-2353(2000)13:4<294::aid-ca10>3.0.co;2-c. 10873222

[8] Mortensen M. , Ivey C. M. , Iida M. , and Woo P. , Superior Thyroid Cornu Syndrome: an Unusual Cause of Cervical Dysphagia, Annals of Otology, Rhinology and Laryngology. (2009) 118, no. 12, 833–838, 10.1177/000348940911801202. 20112516

[9] Browning S. T. , Whittet H. B. , and Williams A. , Failure of Insertion of a Laryngeal Mask Airway Caused by a Variation in the Anatomy of the Thyroid Cartilage, Anaesthesia. (1999) 54, no. 9, 884–886, 10.1046/j.1365-2044.1999.00964.x. 10460563

[10] Welleschik , Larynx Normal2a, 2008, Wikimedia Commons.

[11] Li W. W. and Lou Z. , The Unilateral Excessive Length of Superior Cornu of Thyroid Cartilage: A Case Report, Ear, Nose, and Throat Journal. (2024) 10.1177/01455613241298069. 39540690

[12] Nadig S. K. , Uppal S. , Back G. W. , Coatesworth A. P. , and Grace A. R. , Foreign Body Sensation in the Throat due to Displacement of the Superior Cornu of the Thyroid Cartilage: Two Cases and a Literature Review, Journal of Laryngology and Otology. (2006) 120, no. 7, 608–609, 10.1017/s0022215106001125. 16681864

[13] Lin D. , Fischbein N. , and Eisele D. W. , Odynophagia Secondary to Variant Thyroid Cartilage Anatomy, Dysphagia. (2005) 20, no. 3, 232–234, 10.1007/s00455-005-0012-2. 16362512

[14] Aviv J. E. , Berkower A. S. , and Urken M. L. , Carotid Artery Laceration Secondary to Thyroid Cartilage Fracture: an Unusual Complication of Blunt Neck Trauma, Otolaryngology–Head and Neck Surgery. (1991) 104, no. 3, 375–379, 10.1177/019459989110400315. 1902941

[15] Smith M. E. , Berke G. S. , Gray S. D. , Dove H. , and Harnsberger R. , Clicking in the Throat: Cinematic Fiction or Surgical Fact?, Archives of Otolaryngology—Head and Neck Surgery. (2001) 127, no. 9, 1129–1131, 10.1001/archotol.127.9.1129. 11556866

[16] Cascini F. , Biondo D. , Giannace C. et al., A Fatal Mediastinitis due to a Neck Trauma From an Undeclared Assault, Journal of Forensic Science. (2019) 64, no. 4, 1234–1237, 10.1111/1556-4029.13957. 30444943

[17] Kao C. H. , Wang H. W. , Lee J. C. , and Chin S. C. , Pseudotumor of the Hypopharynx-Displaced Superior Cornu of the Thyroid Cartilage, Otolaryngology–Head and Neck Surgery. (2005) 132, no. 1, 167–168, 10.1016/j.otohns.2004.03.044. 15632937

[18] Redman A. G. , Hide I. G. , and Zammit-Maempel I. , Osteoma of the Thyroid Cartilage—An Unusual Cause of Difficult Intubation, British Journal of Radiology. (2000) 73, no. 872, 899–900, 10.1259/bjr.73.872.11026869. 11026869

[19] Avrahami E. , Harel M. , and Englender M. , CT Evaluation of Displaced Superior Cornu of Ossified Thyroid Cartilage, Clinical Radiology. (1994) 49, no. 10, 683–685, 10.1016/s0009-9260(05)82659-5. 7955828

