# Supplementary material for: An Enlarged Superior Horn of Thyroid Cartilage Presenting During Intubation: A Case Report
Source: Case Rep Anesthesiol. 2026 Aug 23;2026:6068920. doi: 10.1155/cria/6068920 (PMC13501018; doi:10.1155/cria/6068920)
Supplement: Supplementary file 1 [file CRIA-2026-6068920-s001.pdf]

CARE case report guidelines

CARE Checklist of information to include when writing a case report

(cc) BY-NC-ND

✓

Topic	Item	Checklist item description	Reported on Line
Title	1	The diagnosis or intervention of primary focus followed by the words “case report”	1-2
Key Words	2	2 to 5 key words that identify diagnoses or interventions in this case report, including "case report"	Not present on manuscript document- part of Wiley submission
Abstract (no references)	3a	Introduction: What is unique about this case and what does it add to the scientific literature?	25-26
	3b	Main symptoms and/or important clinical findings	30-32
	3c	The main diagnoses, therapeutic interventions, and outcomes	33 "prominent SHTC", as no more formal dx exists
	3d	Conclusion—What is the main “take-away” lesson(s) from this case?	51-53
Introduction	4	One or two paragraphs summarizing why this case is unique (**may include references**)	69-78
Patient Information	5a	De-identified patient specific information.	81-82
	5b	Primary concerns and symptoms of the patient.	88-91
	5c	Medical, family, and psycho-social history including relevant genetic information	86-87
	5d	Relevant past interventions with outcomes	91-93
Clinical Findings	6	Describe significant physical examination (PE) and important clinical findings.	96-97
Timeline	7	Historical and current information from this episode of care organized as a timeline	Past care- 83-86. Current care 104-108
Diagnostic Assessment	8a	Diagnostic testing (such as PE, laboratory testing, imaging, surveys).	94-96, 117-121
	8b	Diagnostic challenges (such as access to testing, financial, or cultural)	98-101
	8c	Diagnosis (including other diagnoses considered)	86-87 (Rule out of traumatic cause)
	8d	Prognosis (such as staging in oncology) where applicable	124-125
Therapeutic Intervention	9a	Types of therapeutic intervention (such as pharmacologic, surgical, preventive, self-care)	175-177
	9b	Administration of therapeutic intervention (such as dosage, strength, duration)	104-107 (Anesthesia, N/A for treatment of anatomic variants)
	9c	Changes in therapeutic intervention (with rationale)	N/A- No treatment performed for anatomic variants
Follow-up and Outcomes	10a	Clinician and patient-assessed outcomes (if available)	113-116 (Anesthesia and decision on treatment of anatomic variants)
	10b	Important follow-up diagnostic and other test results	117-121
	10c	Intervention adherence and tolerability (How was this assessed?)	N/A- No intervention performed to anatomy
	10d	Adverse and unanticipated events	112-113
Discussion	11a	A scientific discussion of the strengths AND limitations associated with this case report	Strengths- 139-142, Limitations- 145-146, 177-179, 189-191, 199-201
	11b	Discussion of the relevant medical literature **with references**.	Anatomy 123-139, Clinical 143-158,173-177
	11c	The scientific rationale for any conclusions (including assessment of possible causes)	149-164
	11d	The primary “take-away” lessons of this case report (without references) in a one paragraph conclusion	182-201
Patient Perspective	12	The patient should share their perspective in one to two paragraphs on the treatment(s) they received	N/A- Anatomic variant was not treated.
Informed Consent	13	Did the patient give informed consent? Please provide if requested	Yes ☒ No ☐

Verbal informed consent obtained during preoperative consultation.
