# Supplementary material for: An Enlarged Superior Horn of Thyroid Cartilage Presenting During Intubation: A Case Report
Source: Case Rep Anesthesiol. 2026 Aug 23;2026:6068920. doi: 10.1155/cria/6068920 (PMC13501018; doi:10.1155/cria/6068920)
Supplement: Supplementary file 2 [file CRIA-2026-6068920-s002.zip › Table 1- CARE Timeline.docx]

Table 1- Patient Care Timeline (CARE)

| Patient Care Timeline (CARE) | |
| --- | --- |
| First cervical procedure (source of CT) | 12/26/2002 |
| Second cervical imaging (source of xray) | 5/29/2021 |
| Sacroiliac fusion (anesthesia event documented by this report) | 9/4/2025 |
| Preoperative discussion |  |
| Patient consent |  |
| Induction (Fentanyl, Lidocaine, Midazolam, Propofol) |  |
| Induction (Succinylcholine) |  |
| Intubation |  |
| Sacroiliac fusion procedure |  |
| Emergence and recovery |  |
| Review of previous imaging | 11/26/2025 |
| Structure identified, case documentation initiated | 12/6/2025 |
